# A "prime-pull" immunotherapy approach using a lentiviral vector and intratumoral TLR4 agonist redirects cytotoxic T cells

**DOI:** 10.1186/2051-1426-2-S3-P190

**Published:** 2014-11-06

**Authors:** Tina C Albershardt, Andrea J Parsons, Patrick Flynn, Peter Berglund, Jan ter Meulen

**Affiliations:** 1 Immune Design, Seattle, WA, USA

## Introduction

The clinical efficacy of tumor specific effector T cells is limited by their proper trafficking to the site of the tumor and the locally immunosuppressive environment. Strategies to improve homing and activity of immune effector cells to tumors are needed to unlock the potential of active cancer immunotherapy.

## Methods

B16F10-OVA melanoma bearing C57BL/6 mice were immunized with the lentiviral vector DCVex on 10 days after tumor challenge to induce OVA specific effector and memory CD8 T cells. Mice were then treated 12 days later intratumorally with the TLR4 agonist GLAAS (glucopyranosyl adjuvant system), which induces the T cell homing chemokines CXCL9 and CXCL10. Control mice received no treatment or only intratumoral GLAAS injection. Tumors were excised, homogenized, and the number and phenotype of OVA-specific infiltrating lymphocytes were analyzed via flow cytometry by staining cells with phenotyping cell surface markers and OVA-pentamers. Analysis was done 0, 24, and 48 hours after GLAAS injection.

## Results

Untreated, B16F10-OVA tumor bearing mice had no evidence of OVA specific CD4 or CD8 T cells by flow cytometry analysis. In contrast, a single injection with DCVex-OVA induced a peak response of 8-9% specific CD8 cells, which were detectable at low levels up to 35 days post-immunization. Intratumoral GLA administration of tumor bearing, DCVex immunized mice resulted in a mean of 7-fold intratumoral increase in OVA specific CD8 T cells, compared to mice treated with GLA only or left untreated.

## Conclusion

Intratumoral injection of the TLR4 agonist GLAAS effectively redirects systemically induced CD8 T cells to the site of the tumor. Because GLAAS also stimulates antigen presentation and maturation of dendritic cells, the prime-pull approach may be a very effective way to overcome two major barriers to active cancer immunotherapy.

